# Supplementary material for: EZH2 and KDM6B Expressions Are Associated with Specific Epigenetic Signatures during EMT in Non Small Cell Lung Carcinomas
Source: Cancers (Basel). 2020 Dec 5;12(12):3649. doi: 10.3390/cancers12123649 (PMC7762040; doi:10.3390/cancers12123649)

Western blots figure 2

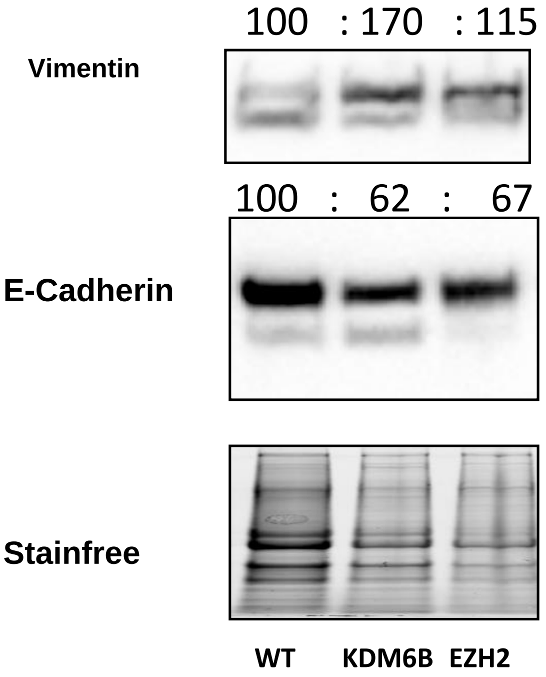

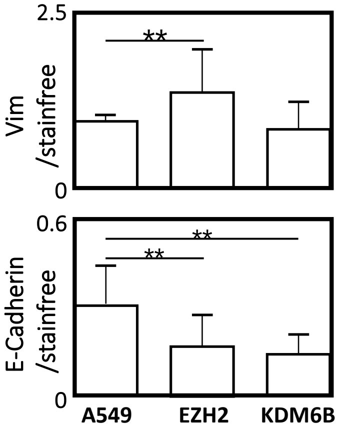

Western blots figure 3

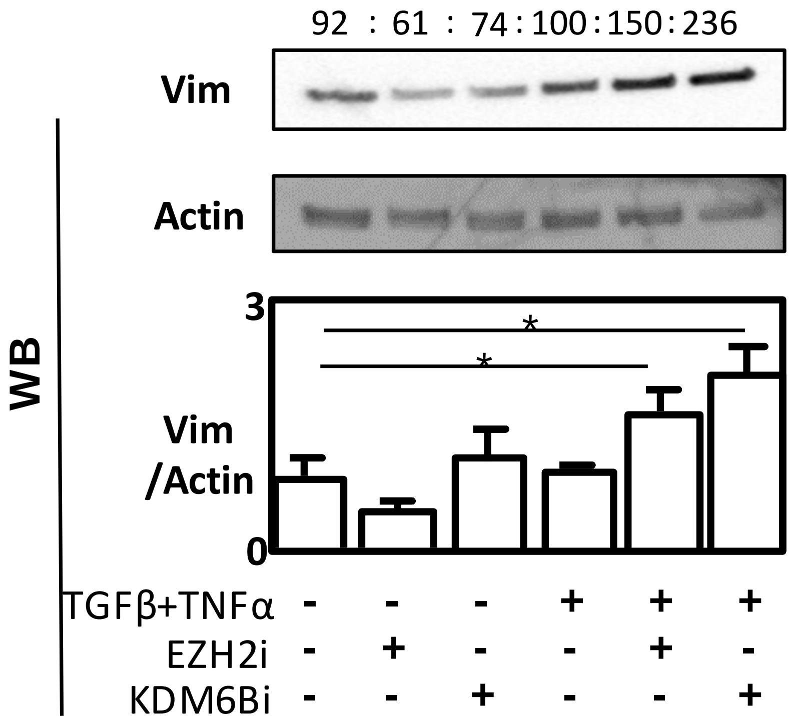

Actin

A

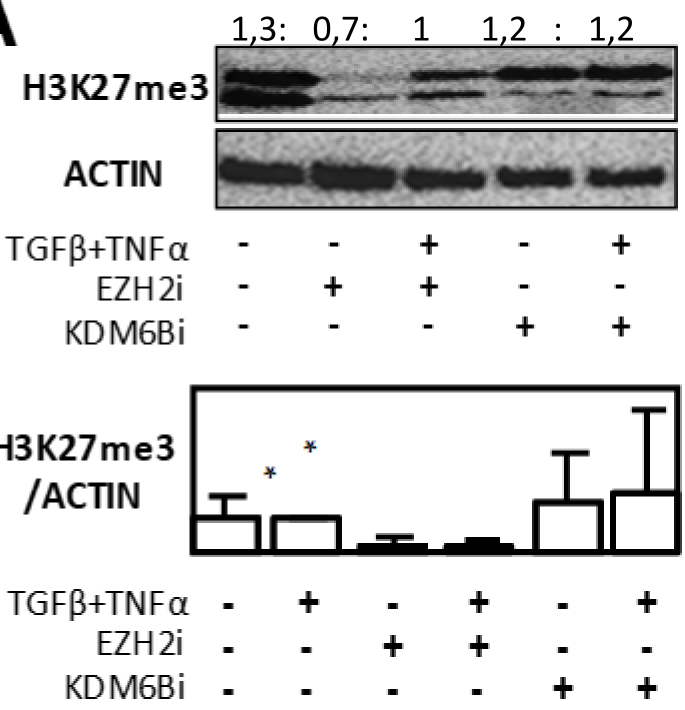

Supp figure 2 :

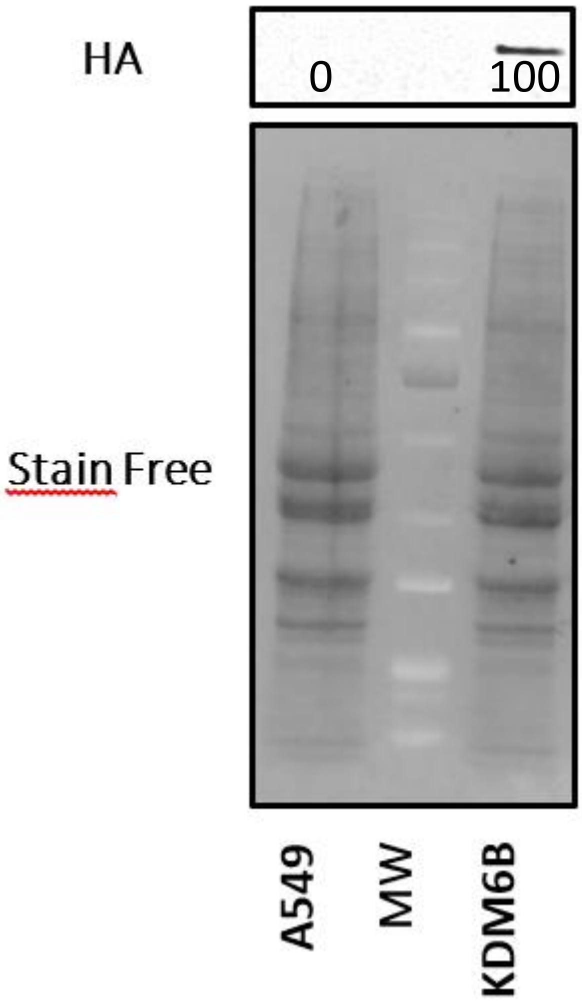

Supp figure 4 :

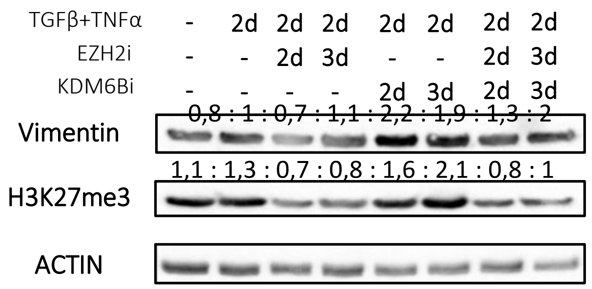

Supp figure 5 :

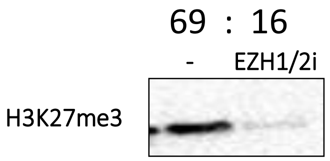

Supp figure 6 :

A

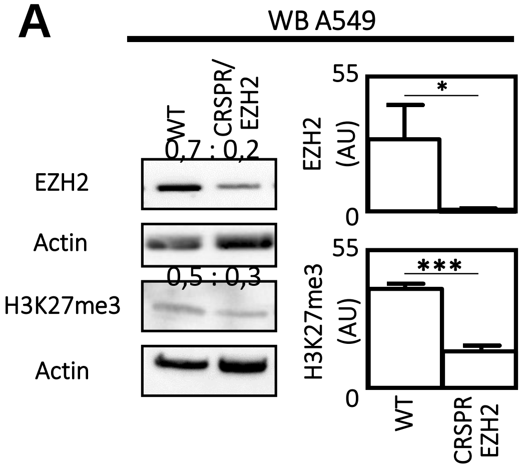

B

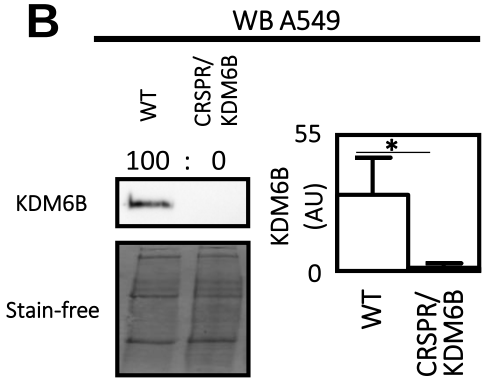

E

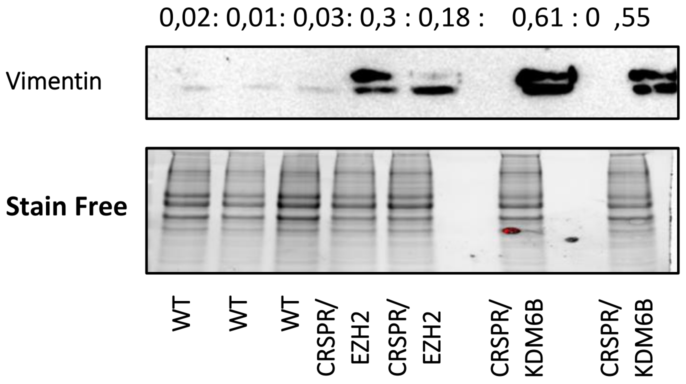

Western blots Figure 2

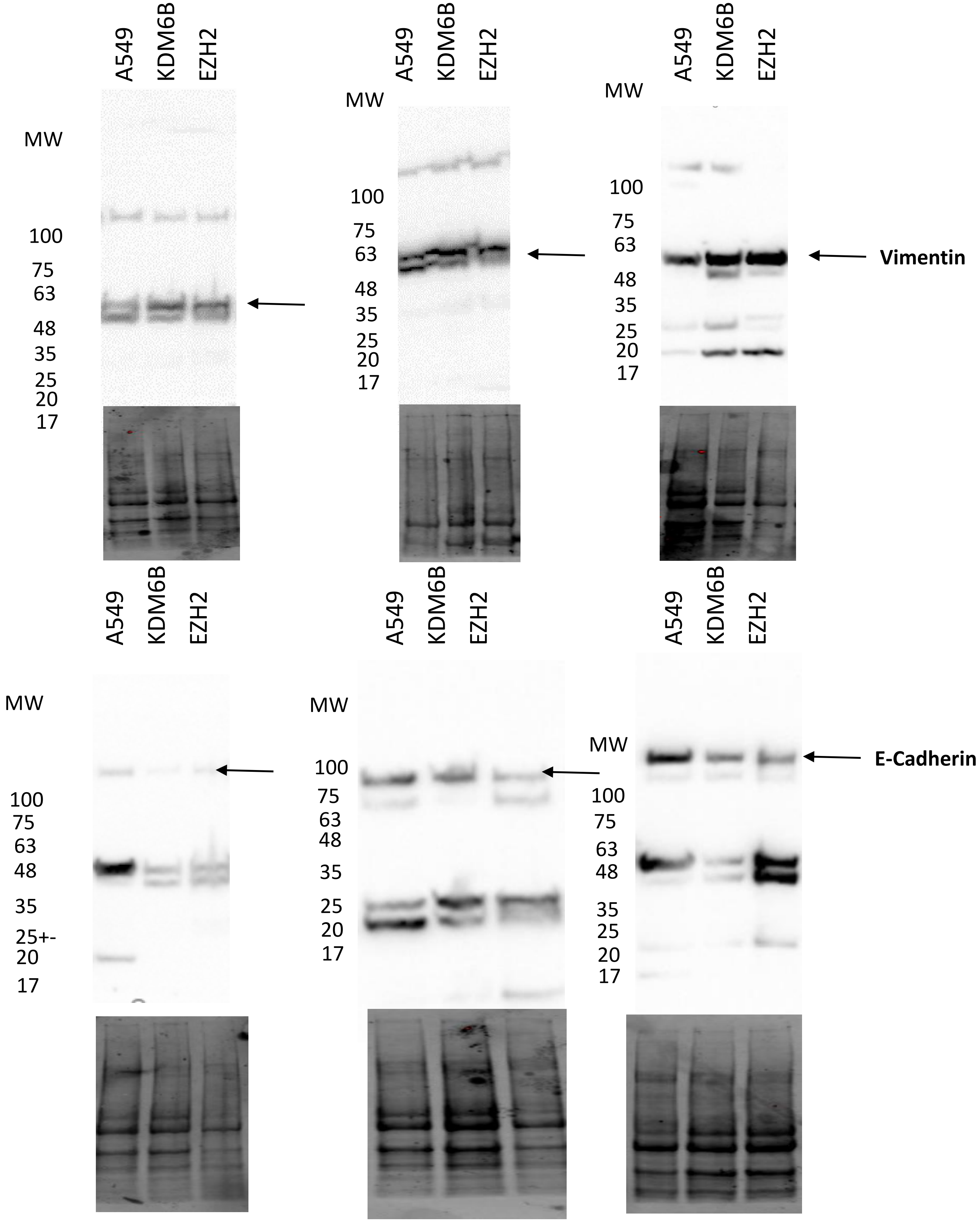

Western blots Figure 3

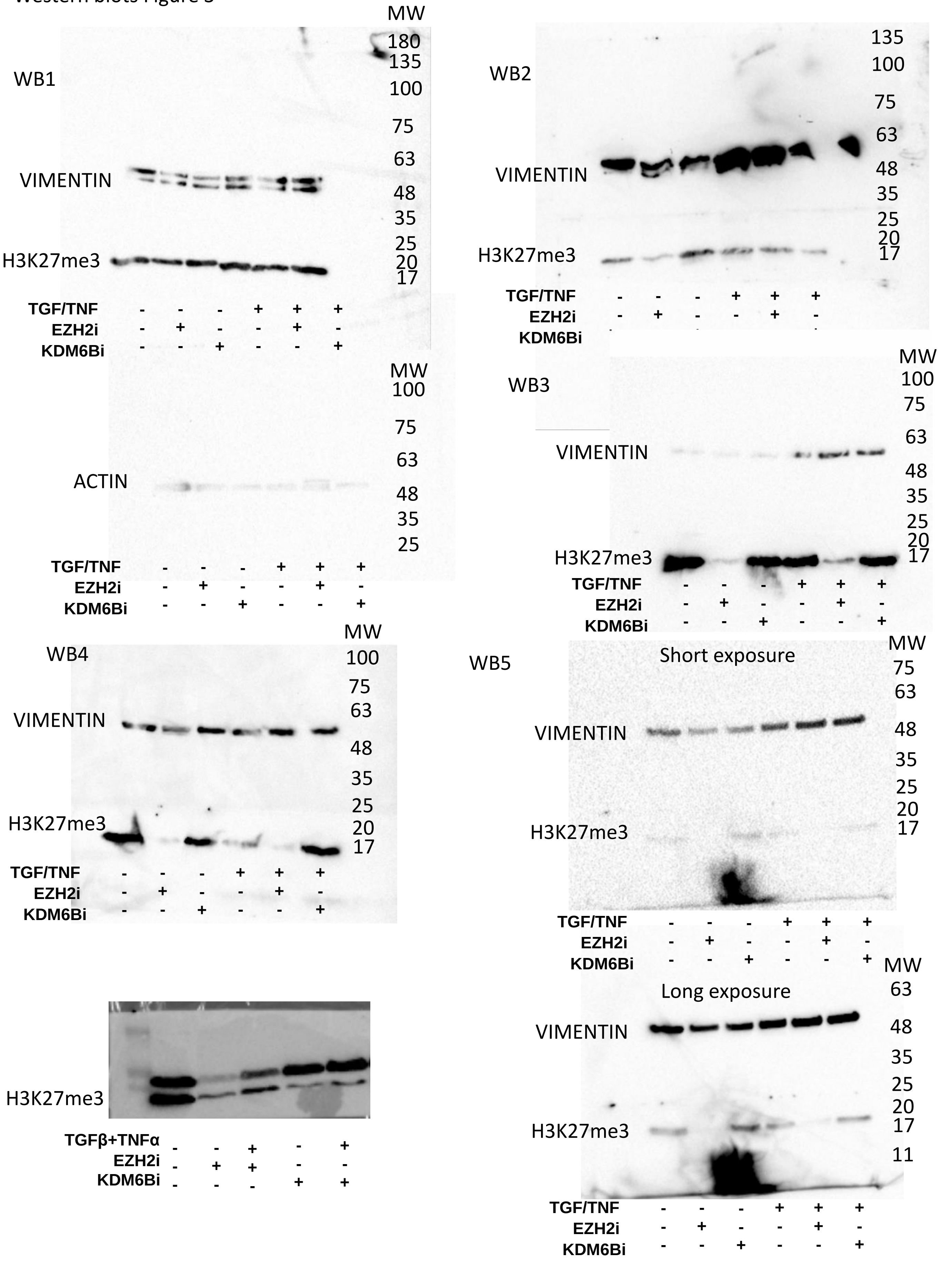

Western blots supp figure 2 : surexp KDM6B

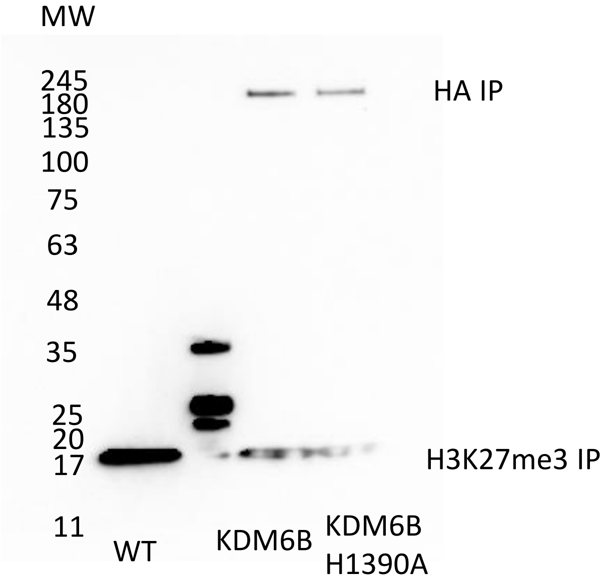

Western blots supp figure 4 : EZH2i and KDM6Bi

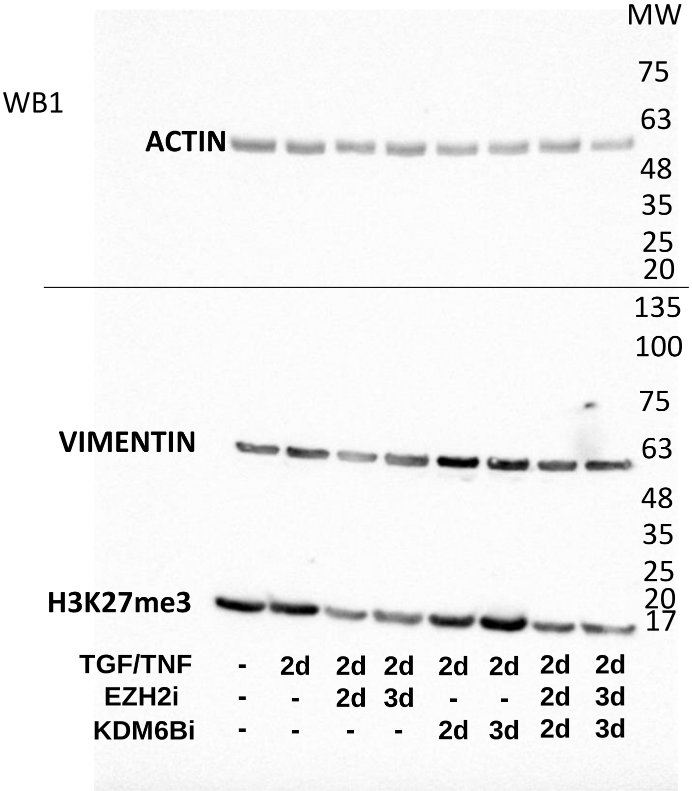

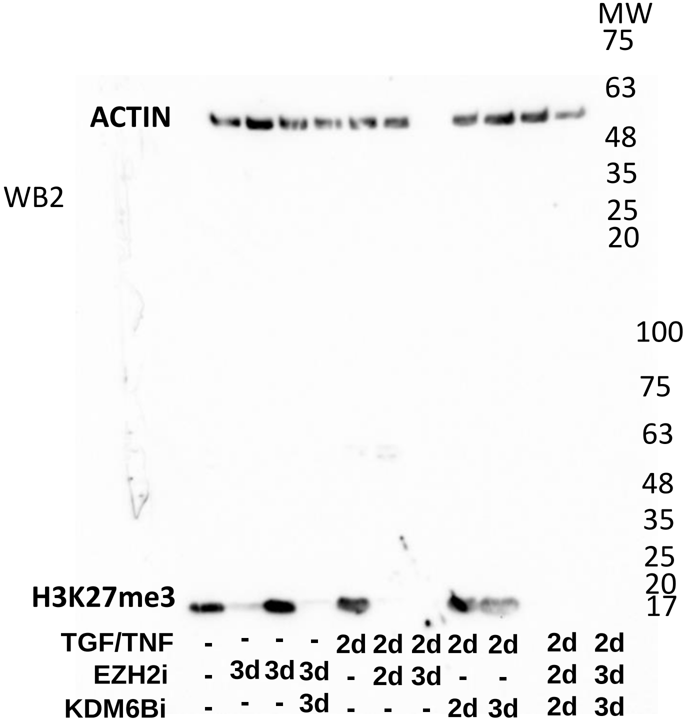

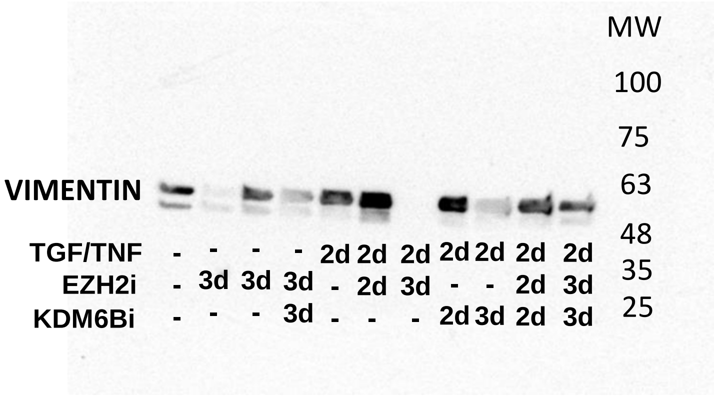

Western blots supp figure 6

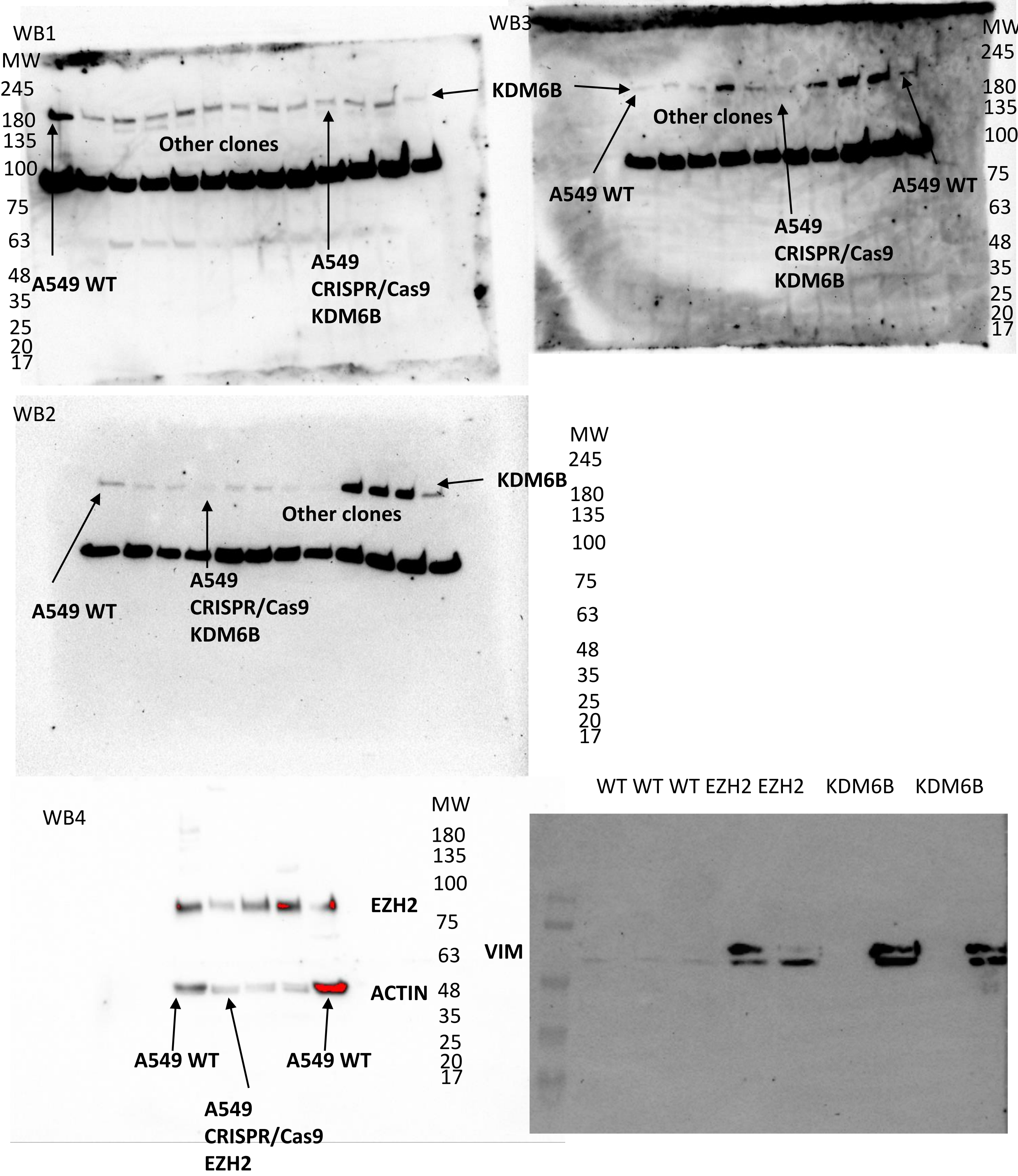

Supplement: Supplementary file 1 [file cancers-12-03649-s001.zip › cancers-998838_supplementary/cancers-998838_raw WB.pdf]
