# Supplementary material for: EZH2 and KDM6B Expressions Are Associated with Specific Epigenetic Signatures during EMT in Non Small Cell Lung Carcinomas
Source: Cancers (Basel). 2020 Dec 5;12(12):3649. doi: 10.3390/cancers12123649 (PMC7762040; doi:10.3390/cancers12123649)
Supplement: Supplementary file 1 [file cancers-12-03649-s001.zip › cancers-998838_supplementary/cancers-998838-supplementary.docx]

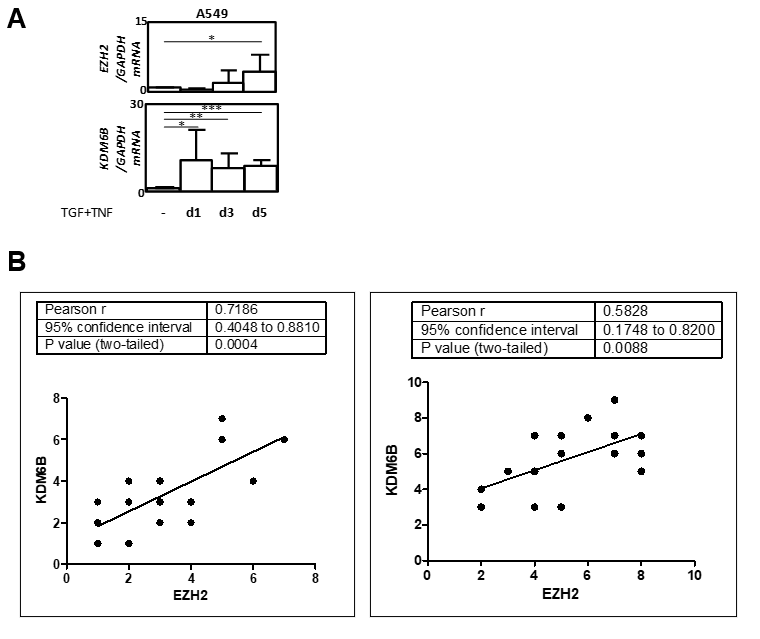


**Figure S1.** Expression of EZH2 and KDM6B during EMT inA549 cellular model and correlation between EZH2 and KDM6B expression in NSCLCs. A) mRNA quantification of *EZH2* and *KDM6B* transiently expressed in A549 cells using RT-qPCR. B) Correlation of EZH2 and KDM6B expression in NSCLCs. Left panel: VIM-negative tumors; Right panel: VIM-positive tumors. * = 0.01<p<0.05; ** = 0.005<p<0.01; *** = 0.001<p<0.0005; **** = p<0.0005.

**
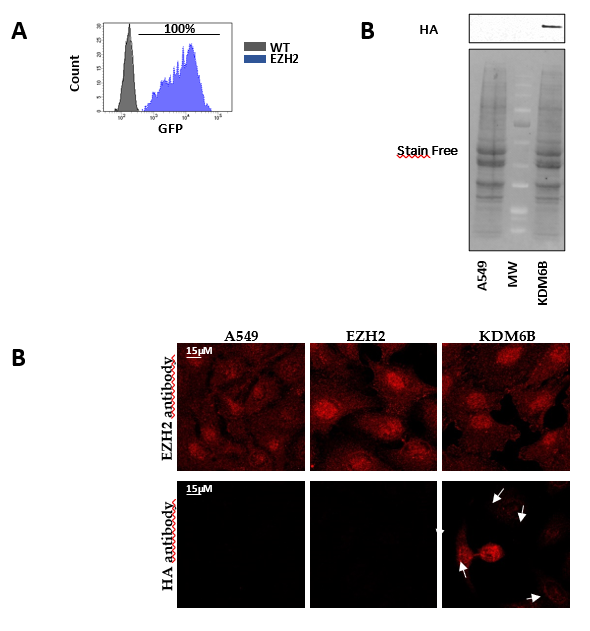
**

**Figure S2. Validation of stable transfection of EZH2 and KDM6B in A549 cells.** **A.** FACS analysis perfomed after selection with puromycin of stably transduced A549 cells with the pMSCV-EZH2-PGK-Puro-438 IRES-GFP vectors. **B**. Validation of overexpression of HA-KDM6B after stable transfection of A549 cells with the pMSCV-KDM6B-HA plasmid by Western-blot analysis. **C**) IF staining of EZH2 and KDM6B in A549 cells stably transfected with the pMSCV-EZH2-PGK-Puro-438 IRES-GFP or pMSCV-KDM6B-HA vector.


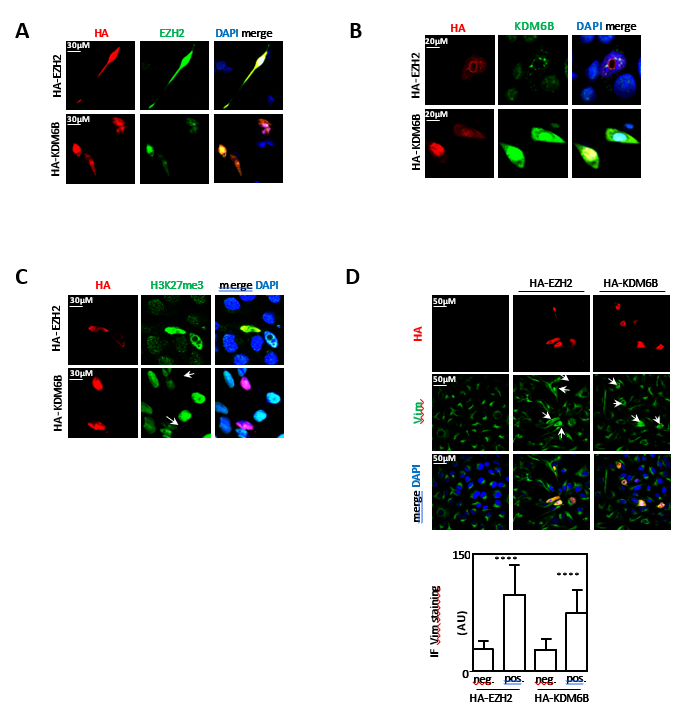


**Figure S3. Effect of transient overexpression of EZH2 and KDM6B on EMT induction.** **A-C)** IF staining of HA (red) and H3K27me3 or EZH2 or KDM6B (green) in A549 cells transiently transfected with HA-EZH2- or HA-KDM6B-expressing plasmids. **D)** Left panel : IF images showing an increased Vimentin staining in A549 cells transiently transfected with HA-EZH2- or HA-KDM6B-expressing vector. Right: Vimentin staining quantification. * = 0.01<p<0.05; ** = 0.005<p<0.01; *** = 0.001<p<0.005; **** = p<0.001.


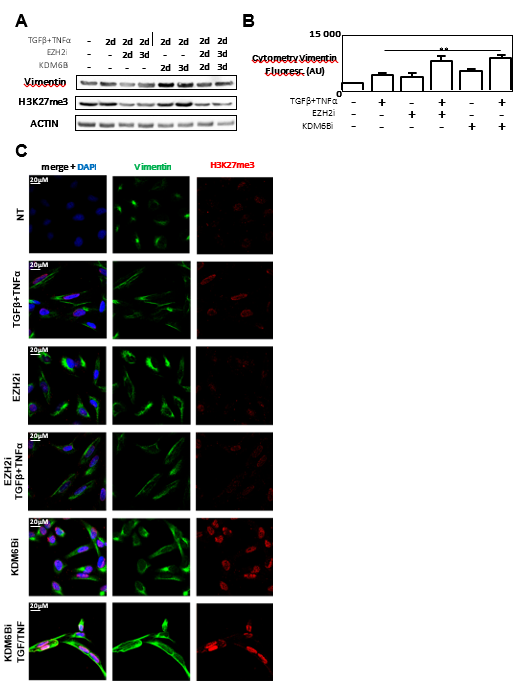


**Figure S4. EZH2i and KDM6Bi regulated the expression of Vimentin and the levels of H3K27me3 in A549 cells.** **A**) Analysis of Vimentin expression and H3K27me3 levels using WB in A549 cells treated with TGFβ/TNFα, and/or EZH2i, and/or KDM6Bi for 2 to 3 days. **B**) Increased Vimentin expression analyzed by flow cytometry following EZH2i or KDM6Bi treatment in the A549 cells. Vimentin expression was quantified using cytometry in A549 cells treated with TGFβ/TNFα, and/or EZH2i, and/or KDM6Bi for 3 days. Ten thousand cells from each sample were evaluated for fluorescence detection using the BD FACSCanto cytometer. (Becton Dickinson, Le Pont de Claix, France) and analyzed using the FACS Diva software. **C**) IF staining of H3K27me3 (red) and Vimentin (green) in A549 cells treated with/without TGFβ/TNFα, and/or EZH2i, and/or KDM6Bi. * = 0.01<p<0.05; ** = 0.005<p<0.01; *** = 0.001<p<0.005; **** = p<0.001.


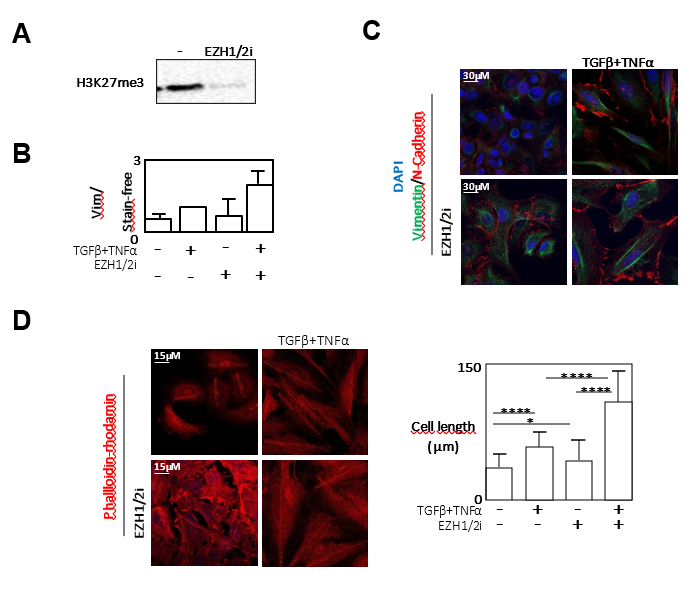


**Figure S5. EZH1/2i (UNC1999) promoted an EMT-related phenotype in A549 cells.** A549 cells were treated with/without TGFβ/TNFα, with/without EZH1/2i for 48 h. **A**) Quantification of H3K27me3 using WB. **B**) Quantification of Vimentin expression after EZH1/EZH2 inhibition in A549 cells treated with or without TGFβ/TNFα. Stain-free staining was used for normalization **C**) Vimentin (green) and N-Cadherin (red) stainings. **D**) Length of A549 cells visualized using a phalloidin-rodhamin staining. * = 0.01<p<0.05; ** = 0.005<p<0.01; *** = 0.001<p<0.005; **** = p<0.001.


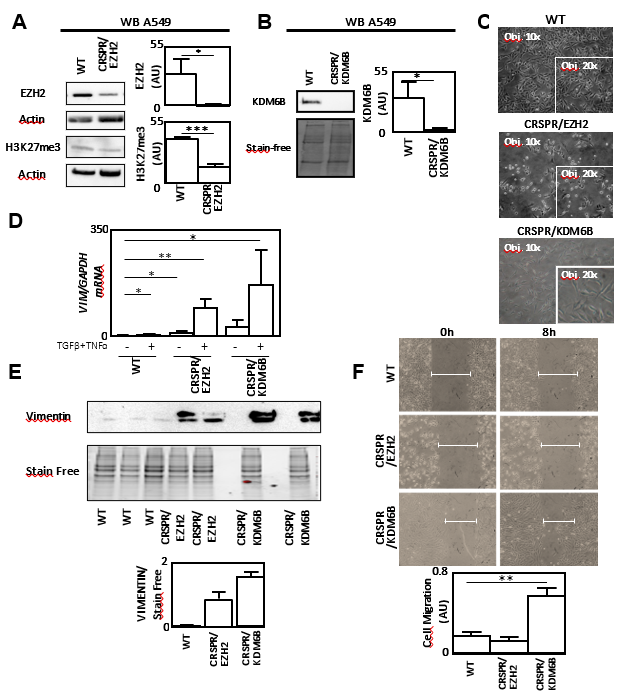


**Figure S6. Invalidation of EZH2 and KDM6B using CRISPR/Cas9 favored EMT-like phenotypes in A549 cells. A)** Validation of decreased EZH2 expression and H3K27me3 content using WB in A549 CRISPR/EZH2. **B)** Validation of decreased KDM6B expression using WB in A549 CRISPR/KDM6B. **C)** Representative pictures of A549, A549 CRISPR/EZH2 and A549 CRISPR/KDM6B cells. **D)** Increased *VIM* expression quantified using qRT-PCR in A549 CRISPR/EZH2 and A549 CRISPR/DM6B cells. **E)** Increased Vimentin expression quantified using WB in A549 CRISPR/EZH2 and A549 CRISPR/KDM6B cells. **F)** Increased migration capacities measured by a wound healing test in A549 CRISPR/EZH2 and A549 CRISPR/KDM6B cells (EVOS™ 4X Objective). * = 0.01<p<0.05; ** = 0.005<p<0.01; *** = 0.001<p<0.005; **** = p<0.001.


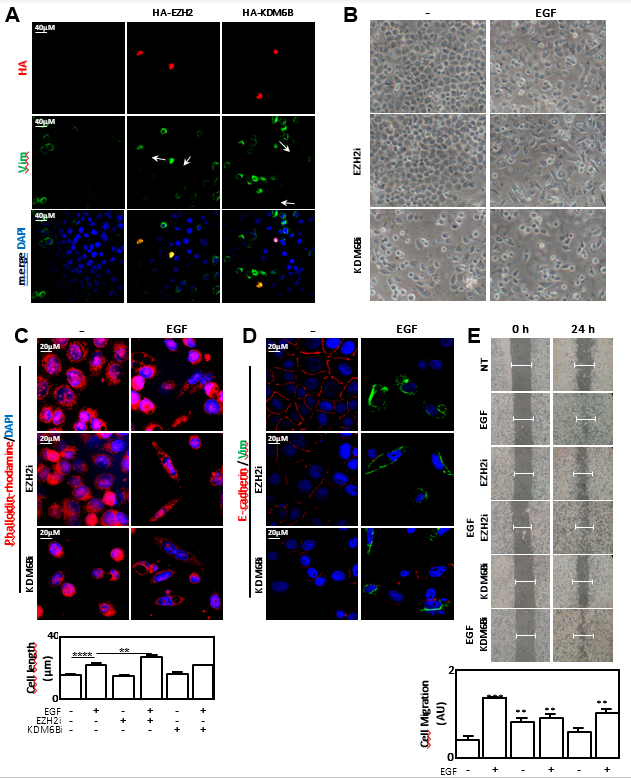


**Figure S7. Overexpression and chemical inhibition of EZH2 and KDM6B regulated EMT-like phenotypes in MDA-MB-468 cells. A**) IF staining of HA (red) and Vimentin (green) in MDA-MB-468 cells transfected with HA-EZH2 or HA-KDM6B-expressing vector. **B**) Representative phenotype of MDA-MB-468 cells treated with EGF, and/or EZH2i, and/or KDM6Bi (EVOS™ 10X Objective). **C**) Upper panel: Phalloidin-rhodamin staining of MDA-MB-468 cells treated with EGF, and/or EZH2i, and/or KDM6Bi. Lower panel: quantification. **D**) IF staining of E-Cadherin (red) and Vimentin (green) in MDA-MB-468 cells treated with EGF, and/or EZH2i, and/or KDM6Bi. **E**) Representative pictures of wound healing experiments in MDA-MB-468 cells treated with EGF, and/or EZH2i, and/or KDM6Bi and quantifications (EVOS™ 4X Objective). * = 0.01<p<0.05; ** = 0.005<p<0.01; *** = 0.001<p<0.005; **** = p<0.001.


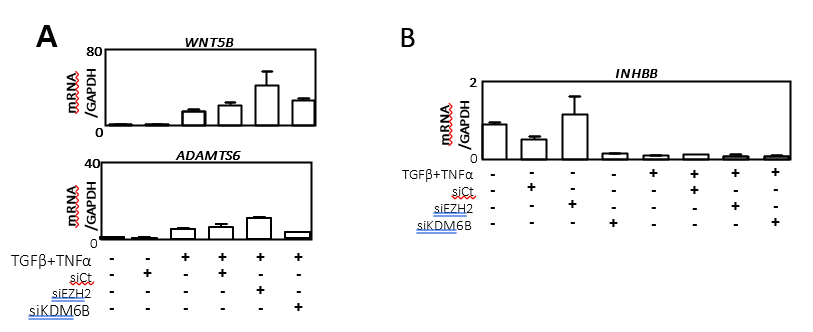


**Figure S8. Effects of siEZH2 or siKDM6Bi on target genes.** A) Modulation of WNT5B, ADAMTS6 and, B) INHBB gene expression in A549 cells following exposure to TGFβ/TNFα treatment and siEZH2 or siKDM6B.

| 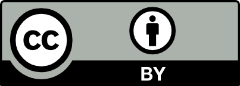 | © 2020 by the authors. Submitted for possible open access publication under the terms and conditions of the Creative Commons Attribution (CC BY) license (http://creativecommons.org/licenses/by/4.0/). |
| --- | --- |
